# Right ventricular myocardial oxygen tension is reduced in monocrotaline-induced pulmonary hypertension in the rat and restored by myo-inositol trispyrophosphate

**DOI:** 10.1038/s41598-021-97470-6

**Published:** 2021-09-09

**Authors:** Marta Oknińska, Zuzanna Zambrowska, Karolina Zajda, Aleksandra Paterek, Klaudia Brodaczewska, Urszula Mackiewicz, Cezary Szczylik, Adam Torbicki, Claudine Kieda, Michał Mączewski

**Affiliations:** 1 Department of Clinical Physiology, Centre of Postgraduate Medical Education, Warsaw, Poland; 2 Laboratory of Molecular Oncology and Innovative Therapies, Military Institute of Medicine, Warsaw, Poland; 3 Department of Oncology, Centre of Postgraduate Medical Education, Warsaw, Poland; 4 Department of Pulmonary Circulation, Thromboembolic Diseases and Cardiology, Centre of Postgraduate Medical Education, Warsaw, Poland; 5 Centre for Molecular Biophysics, CNRS, UPR, 4301 Orléans Cedex 2, France

**Keywords:** Cardiovascular biology, Circulation, Cardiology

## Abstract

Pulmonary hypertension (PH) initially results in compensatory right ventricular (RV) hypertrophy, but eventually in RV failure. This transition is poorly understood, but may be triggered by hypoxia. Measurements of RV oxygen tension (pO_2_) in PH are lacking. We hypothesized that RV hypoxia occurs in monocrotaline-induced PH in rats and that myo-inositol trispyrophosphate (ITPP), facilitating oxygen dissociation from hemoglobin, can relieve it. Rats received monocrotaline (PH) or saline (control) and 24 days later echocardiograms, pressure–volume loops were obtained and myocardial pO_2_ was measured using a fluorescent probe. In PH mean pulmonary artery pressure more than doubled (35 ± 5 vs. 15 ± 2 in control), RV was hypertrophied, though its contractility was augmented. RV and LV pO_2_ was 32 ± 5 and 15 ± 8 mmHg, respectively, in control rats. In PH RV pO_2_ was reduced to 18 ± 9 mmHg, while LV pO_2_ was unchanged. RV pO_2_ correlated with RV diastolic wall stress (negatively) and LV systolic pressure (positively). Acute ITPP administration did not affect RV or LV pO_2_ in control animals, but increased RV pO_2_ to 26 ± 5 mmHg without affecting LV pO_2_ in PH. RV oxygen balance is impaired in PH and as such can be an important target for PH therapy. ITPP may be one of such potential therapies.

## Introduction

Pulmonary hypertension (PH) is a condition involving increased pulmonary vascular resistance (PVR) that results in increased mean pulmonary artery pressure (mPAP) and afterload of the cardiac right ventricle (RV). This initially results in compensatory RV hypertrophy, but when RV is no longer able to compensate for the increased afterload, eventually RV failure occurs resulting in patient’s death^1^.

The cause of this transition from compensatory RV hypertrophy to decompensated RV failure is unclear^2^. Several lines of evidence indicate that impaired RV metabolism^3^ and in particular RV hypoxia, i.e. reduced myocardial oxygen tension, may underlie this phenomenon^4,5^. First, in PH oxygen delivery is impaired due to increased extravascular compression of RV coronary vessels (due to both RV hypertrophy and increased RV pressures) and reduced coronary perfusion pressure (due to reduced aortic pressure as a consequence of reduced LV cardiac output). Second, increased RV afterload results in proportionally increased energy demand. Third, capillary rarefaction was found in various animal PH models as well as in humans^6^. Fourth, these changes are accompanied by adverse metabolic reprogramming^7^. Fifth, increased activation of right ventricular hypoxia-inducible factor-1 (HIF-1) pathway, a marker of tissue hypoxia, was found in various models of PH^8^.

However, direct measurements of RV myocardial oxygen tension are lacking due to technical difficulties. Indirect methods, utilizing e.g. ^1^H NMR techniques which estimate the ratio of deoxygenated to oxygenated myoglobin or magnetic resonance imaging based on differences in magnetic properties between oxygenated and deoxygenated hemoglobin^9^ exhibit poor specificity and spatial resolution, but indeed suggest that RV oxygen partial pressure (pO_2_) may be reduced in humans with PH. However, some studies suggest normal RV oxygen availability^10^, while other studies indicate the presence of so called pseudohypoxia, i.e. activation of hypoxia-dependent molecular pathways without true tissue hypoxia^11^.

Recently *myo-inositol tris-pyrophosphate* (ITPP), a novel membrane-permeant allosteric effector of hemoglobin has been developed to enhance the oxygen release capacity of red blood cells specifically under hypoxic conditions^12^. ITPP lowers the affinity of hemoglobin for oxygen, thus counteracting the effects of hypoxia^13^. We have previously shown that ITPP shifts the oxygen dissociation curve downward, therefore increasing tissue oxygen delivery, increases oxygen delivery to hypoxic tumors^14^, and reduces HIF-1 expression by vascular endothelial cells^15^. This is accompanied by a potent anti-cancer effect in various tumor models in the mouse^14,16^. Moreover, we have recently shown that chronic ITPP treatment partially prevents post-myocardial infarction heart failure in the rat model^17^. However effects of ITPP on myocardial pO_2_ have never been studied.

Thus the aim of our study was to verify the hypothesis that RV is hypoxic in the rat model of monocrotaline (MCT)-induced PH and that acute ITPP administration is able to relieve it. For this purpose we used a specific fluorescence-based method to directly measure myocardial pO_2_ in situ in PH and assess the effects of ITPP treatment.

## Methods

Forty four male Wistar rats, weighing 180–220 g, were used. All study animals were used in compliance with local and institutional regulations. The study conformed to the Guide for the Care and Use of Laboratory Animals, US National Institutes of Health (NIH Publication No. 85–23, revised 1996) and was approved by the local ethics committee (Second Warsaw Local Ethics Committee for Animal Experimentation). The manuscript follows the recommendations in the ARRIVE guidelines. ITPP was a kind gift from professor Jean-Marie Lehn, UNISTRA, University of Strasbourg to CK.

### Study protocol

At the age of 4–5 weeks the rats received a subcutaneous injection of MCT (60 mg/kg, Sigma) to induce pulmonary hypertension (n = 26) or saline (n = 18) as a control group. Twenty four days later the animals from both control (n = 13) and MCT (n = 14) groups were anesthetized using inhaled 2% isoflurane, underwent echocardiographic examination, followed by hemodynamic evaluation and pO_2_ measurements. Thereafter they were euthanized and their lungs were processed for histological examination. This time point (day 24) was selected to provide approximately 25% mortality in MCT group, indicating severe PH.

The remaining animals from the control (n = 5) and MCT (n = 5) groups underwent echocardiographic examination, received a single intraperitoneal dose of ITPP (1.5 g/kg b.wt.) and 1 h later were re-anesthetized, underwent echocardiographic examination, followed by hemodynamic evaluation and pO_2_ measurements. Thereafter they were euthanized and their lungs were processed for histological examination. A total of 7 rats died in the MCT group, while there were no deaths in the control group.

### Echocardiographic evaluation

Transthoracic echocardiography was performed using E-cube 15 Platinum (Alpinion Medical Systems) with 17 MHz linear transducer under a light isoflurane sedation. The rats were placed on a heating pad to sustain proper body temperature. Images of the parasternal short-axis view at the papillary muscle level, left and right parasternal long axis view to visualize LV and RV, respectively, and the apical 4-chamber view were recorded with careful attention to obtain high frame rate. LV and RV fractional shortening as well as LV and RV diastolic and systolic wall thickness were assessed using the M-mode in parasternal long axis view. All measurements were obtained by one observer blinded to the study groups.

### Hemodynamic evaluation

As reported previously^18^, rats were put on a heating pad, anesthetized with 2% isoflurane (oxygen was used as a carrier gas, flow rate 0.5 l/min, providing fraction of inspired oxygen [FiO_2_] of 40%), intubated and put on an animal ventilator. The upper abdominal cavity was opened and the heart was exposed through cutting of the diaphragm. The LV and subsequently RV apex was punctured with a 25G needle and a microtip pressure–volume (PV) catheter (SPR-838, Millar Instruments; Houston, TX) was inserted into the LV, RV, and subsequently pulmonary artery. Its position was established based on pressure and volume signals. After stabilization for 5 min, the signals were continuously recorded at sampling rate of 1000/s using an ARIA P–V conductance system (Millar Instruments) coupled to a PowerLab/4SP A/D converter (AD Instruments; Mountain View, CA) and a personal computer. To characterize cardiac function, first the inferior vena cava was compressed for 10 s and then released to achieve reduction and augmentation of venous return and cardiac preload, respectively. Heart rate, maximal LV and RV systolic pressure (ESP), end-diastolic pressure (EDP), maximal slope of systolic pressure increment (+ dP/dt *max*) and diastolic pressure decrement (− dP/dt *max*), ejection fraction (EF), end-diastolic volume (EDV), end-systolic volume (ESV), stroke volume (SV), and cardiac output (CO) were computed using a cardiac P–V analysis program (PVAN3.2, Millar Instruments). Mean pulmonary artery pressure (mPAP) was calculated as 0.61 × systolic PAP + 2 mmHg. Pulmonary vascular resistance (PVR) was calculated as (mPAP—left atrial pressure)/CO. Indexes of contractility and stiffness [slope of end-systolic and end-diastolic P–V relations (ESPVR and EDPVR)] were also calculated using PVAN3.2. The volume signal was calibrated using serial dilutions of sodium chloride, as recommended by the manufacturer.

### pO_2_ measurements

Myocardial oxygen tension (pO_2_) was measured using a fiberoptic oxygen-sensing device, the OxyLite Pro pO_2_ monitor (Oxford Optronics Ltd., Oxford, UK). This device measures pO_2_ by determining the oxygen-dependent fluorescent lifetime of ruthenium chloride. Probes (100 µm in diameter) are supplied precalibrated by the manufacturer. The tip of an optical fiber probe is covered with ruthenium chloride, which fluorescence lifetime is O_2_-dependent and is inversely proportional to the pO_2_ in the tissue at the circular area with a diameter of 230 µm around the tip. Following excitation by a flash of green light, the measured half-life of the phosphorescence signal can be quantitatively related to the oxygen tension.

The rats were anesthetized using inhaled isoflurane (2%) and connected to a ventilator. The heart was exposed by left-sided thoracotomy, and a 20-gauge needle was used to pierce the epicardium and guide the probe into the myocardium immediately beneath the epicardium. A 100-µm diameter sensing tip was inserted into three locations in the cardiac RV and LV as well as in the liver and spleen. The pO2 signal was recorded until the signal was stable. Only good quality signals were analyzed (defined as at least 3 consistent non-zero pO_2_ measurements, differing by no more than 20% from the mean result). Subsequently data were averaged for each location. The measurements were performed at two FiO_2_ values: standard 40% and 100%.

### Histological lung analysis

The lungs were inflated with OCT (Optimal Cutting Temperature) compound and then placed in plastic molds filled with OCT and snap-frozen in liquid nitrogen. All tissues were maintained at − 80 °C until further analysis.

The representative lung parts were cut into 5 µm-thick sections using Cryostat Microm HM550 (Thermo Fisher Scientific, Massachusetts, USA) and stained with the hematoxylin and eosin (H&E) stain for microscopic analysis. Arterioles with an external diameter of 30–90 µm were assessed and % wall thickness was expressed as (2 × wall thickness/external vessel diameter).

### Statistical analysis

Shapiro–Wilk test was used to test normality of data distribution. Homogeneity of variances was tested by Bartlett’s. Normally distributed data were expressed as means ± SEM. One-way ANOVA was used to test differences between groups. Tukey post hoc test was used to compare data pairs. Non-normally distributed data were presented as median ± inter-quartile ranges + outliers. Subsequently statistical analysis of differences was tested using non-parametric methods (Mann–Whitney test to compare two groups or Kruskal–Wallis ANOVA to compare three or more groups followed by Dunn's post hoc test). Pearson correlation analysis was used to analyze the correlations. Differences were considered significant when *P* < 0.05. The primary end-point of our study was reduction of pO_2_ in RV in MCT vs. control rats. The study had 94% power to detect at least 25% reduction of pO_2_ in RV in MCT versus. control rats if 11 rats were included in each of the study groups. Statistical analyses were performed using SigmaPlot version 14.

## Results

### Pulmonary hypertension: the model

**Figure 1:**
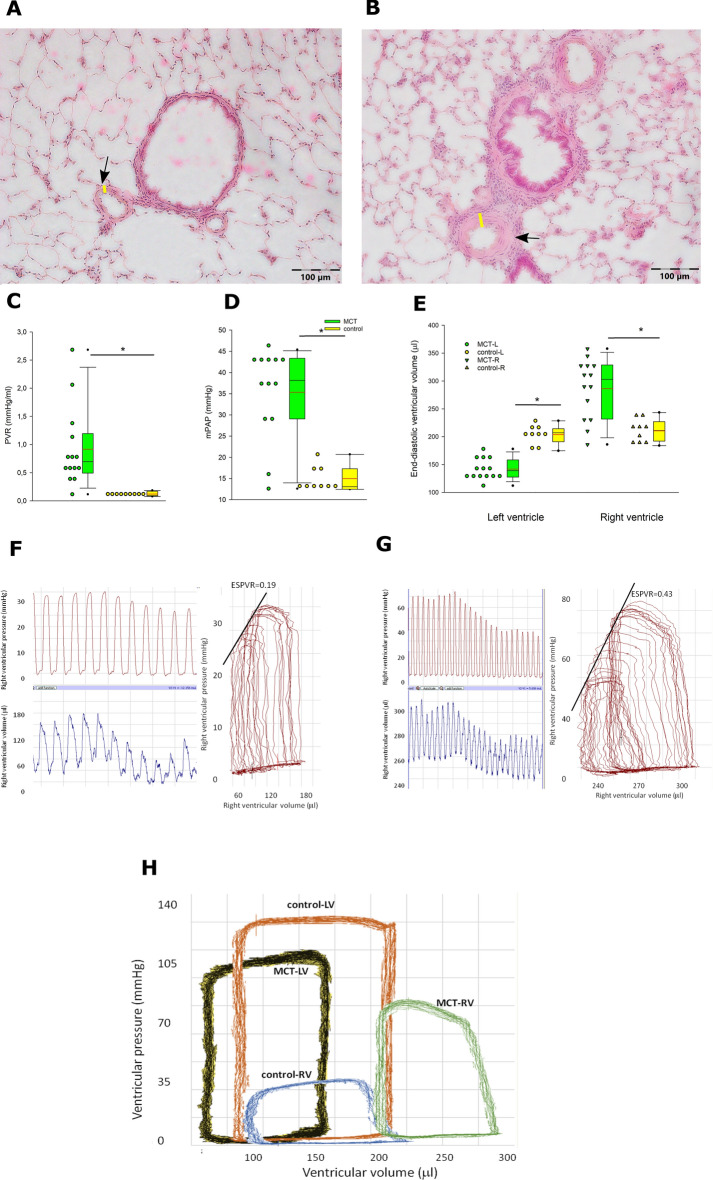
Histological and hemodynamic characterization of rats with monocrotaline-induced pulmonary hypertension and control rats. Representative hematoxylin and eosin stained images from control (**A**) and monocrotaline (MCT)-treated (**B**) rats, demonstrating small pulmonary arterioles (arrows). Yellow bars indicate arteriolar wall thickness. Clear wall thickening and luminal stenosis can be seen in a MCT rat. (**C**) demonstrates pulmonary vascular resistance (PVR), (**D**) mean pulmonary artery pressure (mPAP), and (**E**) left and right ventricular end-diastolic volumes. Representative right ventricular pressure–volume loops and tracings of right ventricular pressure and volumes are presented for control (**F**) and MCT (**G**) rats. (**H**) presents representative RV and LV PV loops from control and MCT rats. Horizontal lines represent mean (red) and median (black), the bottom and the top of the boxes represent the upper and the lower quartile, he whiskers represent 10th and 90th percentile, while the solid circles represent the individual data points. (**C**–**E**) control n = 9; MCT n = 14; since the data exhibited non-normal distribution, the groups were compared using Mann–Whitney test; * *p* < 0.05.

Twenty four days after MCT injection rats demonstrated significant hypertrophy of pulmonary arterioles (% wall thickness 23.4 ± 5.0% vs. 16.7 ± 4.5% in control rats, *p* < 0.05; Fig. 1A,B) and consequently increased pulmonary vascular resistance (PVR, Fig. 1C) and mean pulmonary artery pressure (mPAP, Fig. 1D), revealing typical chain of events for PH.

**Figure 2:**
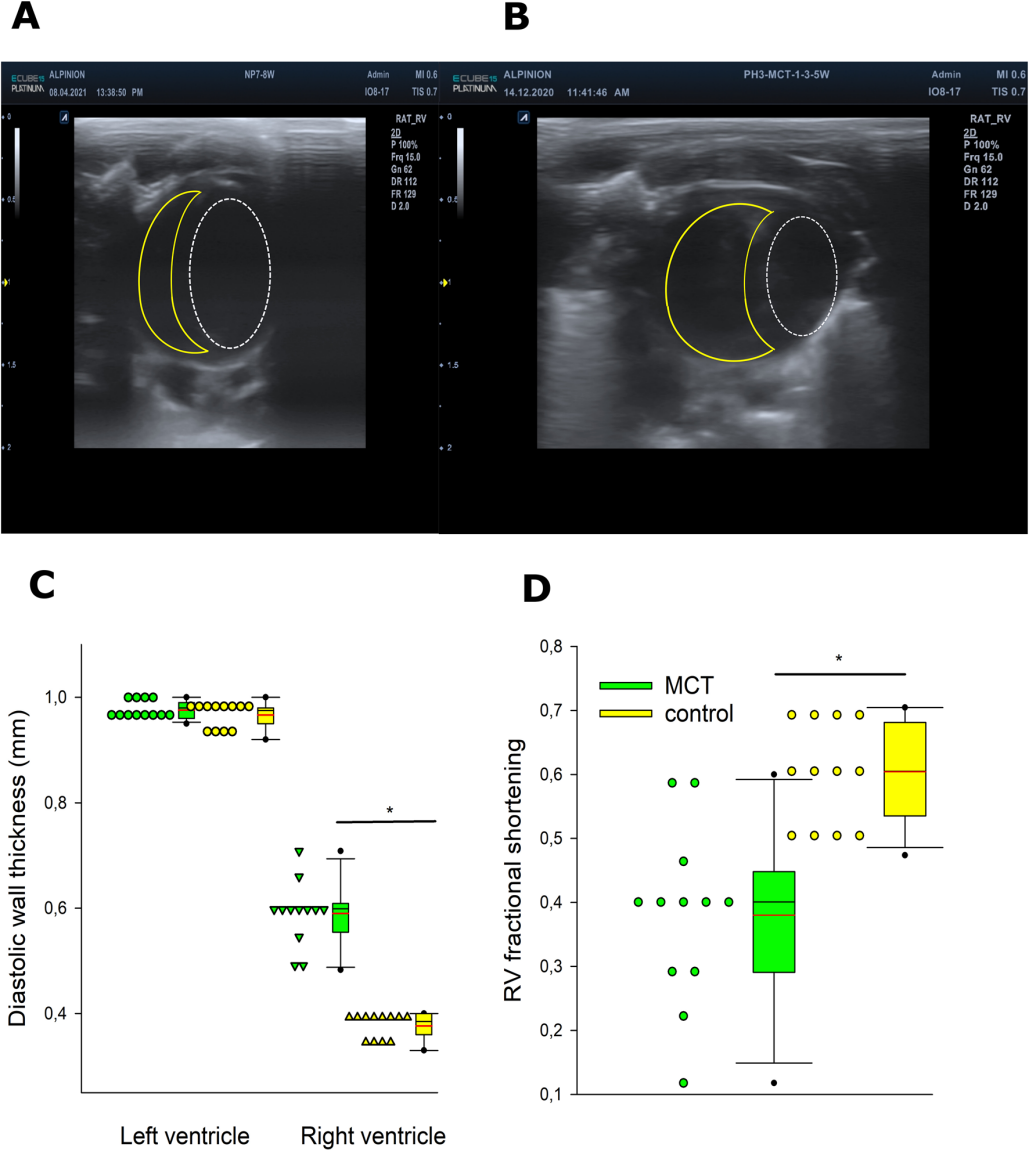
Echocardiographic characterization of rats with monocrotaline-induced pulmonary hypertension and normal rats. Short-axis view of the rat heart in control (**A**) and monocrotaline-treated (**B**) rats. Yellow line outlines contours of the right ventricular cavity, white line of the left ventricular cavity. Markedly dilated right ventricle and compressed left ventricle can be seen in monocrotaline-treated rats (**B**). Lower panels demonstrate right diastolic wall thickness (**C**) and ventricular fractional shortening (**D**). Horizontal lines represent mean (red) and median (black), the bottom and the top of the boxes represent the upper and the lower quartile, he whiskers represent 10th and 90th percentile, while the solid circles represent the individual data points. Control n = 12; MCT n = 12; since the data exhibited non-normal distribution, the groups were compared using Mann–Whitney test;* *p* < 0.05.

RV was dilated, which was reflected by increased RV end-diastolic volume (RVEDV, Figs. 1E–H, 2A,B), while LVEDV was reduced (Figs. 1E–H, 2A,B). Normalized right ventricular (RV) mass (Table 1) and RV diastolic wall thickness (Fig. 2C) were increased by 50%, while the corresponding parameters for LV were unchanged in PH rats.

**Table 1:** Morphological and hemodynamic characteristics of monocrotaline- and saline-treated rats.

	Control (n = 13)	SD	MCT (n = 14)	SD	*p* < 0.05		Control (n = 13)	SD	MCT (n = 14)	SD	*p* < 0.05
Body weight, g	341	10	305	12	*	Normalized					
Heart weight, g	0.94	0.05	0.96	0.06		g/kg	2.76	0.21	3.15	0.24	
LV weight, g	0.61	0.02	0.51	0.03	*	g/kg	1.79	0.06	1.67	0.11	
RV weight, g	0.19	0.02	0.25	0.02	*	g/kg	0.56	0.08	0.82	0.09	*
RV/LV	0.31	0.03	0.49	0.03	*						
Lung weight, g	2.30	0.38	3.04	0.34	*		6.74	1.11	9.97	1.16	*
Left ventricle						Right ventricle					
HR (bpm)	450	18	411	25			444	22	408	26	
CO (mL/min)	43.485	5.447	34.527	2.732	*		47.185	8.329	29.504	4.073	*
EF (%)	59.9	2.1	65.7	2.7			53.9	1.3	27.4	4.1	*
ESP (mmHg)	119.8	2.2	86.0	16.7	*		27.3	1.5	71.5	3.6	*
EDP (mmHg)	7.6	1.9	5.4	0.2			6.0	0.0	9.5	1.0	*
dp/dtmax (mmHg/s)	10,306	1776	8727	1300			2289	79	3074	124	*
dp/dtmin (mmHg/s)	− 6620	946	-4026	854	*		− 1250	154	-2047	94	*
ESPVR (mmHg/µl)	1.32	0.11	1.31	0.12			0.17	0.02	0.48	0.04	*
Ea (mmHg/µl)	0.98	0.07	0.95	0.06			0.23	0.04	0.70	0.11	*
ESPVR/Ea	0.74	0.07	0.74	0.07			0.74	0.09	0.69	0.09	
Diastolic wall stress (mmHg)	9.85	1.56	6.24	1.11	*		8.94	1.43	19.91	9.92	*
Systolic wall stress (mmHg)	168.68	11.84	105.76	26.75	*		36.68	3.47	125.24	51.42	*

CO cardiac output; Ea, arterial elastance; EF, ejection fraction; ESP, end-systolic pressure; EDP, end-diastolic pressure; ESPVR, end-systolic pressure–volume relationship; LV, left ventricle; RV, right ventricle; HR, heart rate.

Ventricular function was characterized using echocardiography and pressure–volume (PV) loops. RV fractional shortening (FS, Fig. 2D) and ejection fraction (EF, Table 1) were impaired and RV PV loops were shifted to the right (Fig. 1E–H). Consequently cardiac output was reduced by 29% (Table 1).

However, maximum rate of RV pressure increase (RV dP/dt *max*) was slightly increased, while the most precise and load-independent index of contractility, slope of end-systolic pressure–volume relation (ESPVR) was increased by almost threefold (Table 1), indicating that RV contractility was actually augmented in our model and impairment of EF and FS was due to increased afterload. RV hypertrophy was not sufficient to prevent increase of RV wall stress: the diastolic wall stress was more than doubled, while the systolic wall stress—more than tripled (Table 1).

On the other hand, LV was unloaded and a result of reduced RV function, resulting in leftward and downward shift of LV PV loops (Fig. 1H) and a low grade reduction of LV diastolic and systolic wall stress (Table 1).

### Myocardial oxygen tension

**Figure 3:**
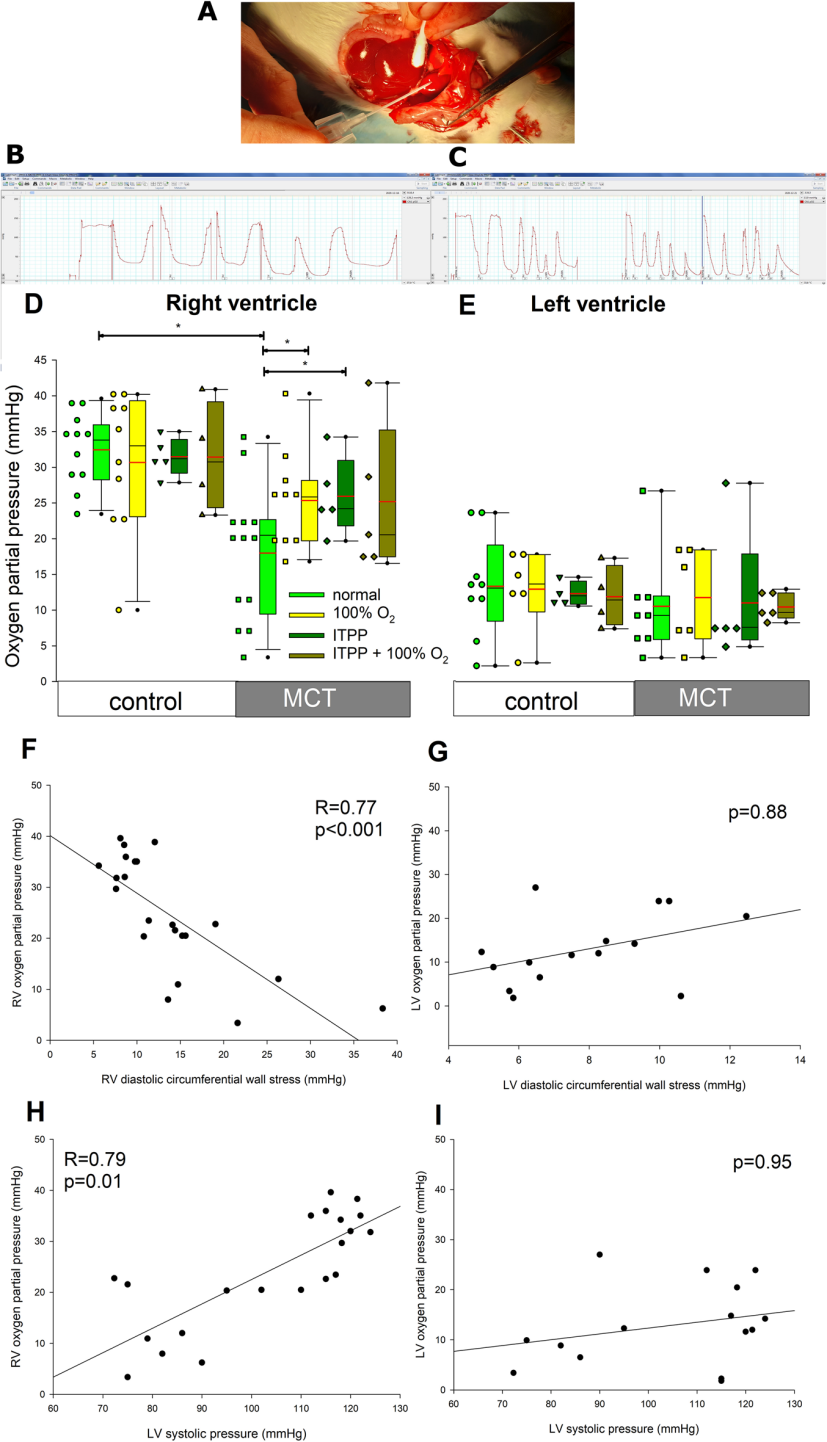
Measurements of myocardial oxygen tension. (**A**) An image demonstrating measurement of right ventricular (RV) partial oxygen pressure (pO_2_) using a fiber-optic probe inserted into the RV, almost parallel to the epicardial surface. Representative recordings of pO_2_ measurements in control and monocrotaline-treated rats are presented on panels (**B**) and (**C**), respectively. The horizontal axis indicates pO_2_. Panel B presents pO_2_ values measured under 40% fraction of inspired oxygen (NORMAL O2), while panel C presents pO_2_ values measured under 40% fraction of inspired oxygen (NORMAL O2) and 100% fraction of inspired oxygen (HIGH O2). RV, right ventricle; LV, left ventricle. The results are summarized for the right and left ventricle on panels (**D**) and (**E**), respectively. Correlations between RV and LV pO_2_ and wall stress as well as LV systolic pressure are shown on panels (**F**) through (**I**). Horizontal lines represent mean (red) and median (black), the bottom and the top of the boxes represent the upper and the lower quartile, he whiskers represent 10th and 90th percentile, while the solid circles represent the individual data points. Control n = 11; MCT = 13. Kruskal–Wallis ANOVA was used to compare the groups followed by Dunn's post hoc test. Pearson correlation analysis was used to analyze the correlations.* *p* < 0.05.

Mean myocardial pO_2_ was 32 ± 5 mmHg and 15 ± 8 mmHg in RV and LV (Fig. 3A–D), respectively, in the control animals, while that in the liver and spleen was 19 ± 11 and 15 ± 11 mmHg, respectively. Breathing with 100% oxygen did not affect these values (Fig. 3A–D).

In PH animals mean RV pO_2_ was reduced by 44% to 18 ± 9 mmHg, while LV pO_2_ was unchanged (11 ± 8 mmHg). pO_2_ values in the liver and spleen were not affected (14 ± 8 and 23 ± 11 mmHg, respectively). Breathing with 100% oxygen increased pO_2_ values in RV to 25 ± 5 mmHg without affecting LV pO_2_ values.

Myocardial oxygen delivery depends on 2 crucial factors, perfusion pressure and vascular resistance. Therefore, to gain insight into potential causes of this reduction of RV pO_2_ we correlated myocardial pO_2_ with LV systolic pressure (LVSP, as a measure of aortic pressure, supplying perfusion pressure to coronary arteries) and with RV wall stress (as a marker of external vascular compression). As Fig. 3F,H show, RV pO_2_ was highly negatively correlated with RV diastolic wall stress and positively correlated with LVSP. No such correlation was found for the LV pO_2_ values (Fig. 3G,I).

### Effect of ITPP

In additional experiments (n = 5) a single dose of ITPP was given intraperitoneally and 1 h later pO_2_ measurements were performed.

ITPP had no effect on pO_2_ values in the control animals: both LV and RV (Fig. 3D) as well as hepatic and splenic (not shown) oxygen tensions were not affected. 100% oxygen breathing had no effect in this setting, either. However, in PH animals, RV pO_2_ was significantly increased by acute ITPP administration by 44% to 26 ± 5 mmHg. ITPP did not affect pO_2_ values in the LV (Fig. 3E) or spleen or liver (not shown). 100% oxygen breathing did not affect RV pO_2_ values in MCT-treated rats that received ITPP. ITPP treatment had no effect on either echocardiographic or hemodynamic parameters (not shown).

## Discussion

In this paper we reveal two new crucial facts regarding myocardial oxygen partial pressure in a rat model of monocrotaline-induced PH: (1) we demonstrate that PH is associated with reduced RV pO_2_ and that this reduction correlates with both reduced LV systolic pressure and increased RV diastolic wall stress and that (2) a new agent, ITPP, that increases oxygen dissociation from hemoglobin, improves RV pO_2_ in PH without affecting it in the healthy RV or LV.

### RV function and structure in our pulmonary arterial hypertension model

In our model of MCT-induced PH, RV exhibited augmented contractile function as manifested by more than threefold increase of ESPVR and marked hypertrophy and dilation corresponding to a compensated stage of RV hypertrophy required to overcome more than fourfold increase of RV afterload. However, this was accompanied by reduction of cardiac output that in turn reduced LV preload and its output, resulting in decreased LV pressures and afterload. Thus, paradoxically RV wall stress increased, LV wall stress decreased and RV wall stresses were higher than those in LV, unlike in normal hearts.

### RV myocardial oxygen tension

Here we showed that RV pO_2_ was more than twofold higher than LV pO_2_ (32 ± 5 mmHg and 15 ± 8 mmHg, respectively) in control rats. These values for LV are within the ranges reported by other authors and obtained using different methods: 45 mmHg in the LV of humans undergoing bypass surgery using voltammetric microelectrode technique^19^, 35 mmHg of mitochondrial O_2_ in the rat LV^20^, 10 mmHg in the rat LV^21^ and 8.6 mmHg in the swine LV using electron paramagnetic resonance oximetry^22^. These results indicate that LV pO_2_ values in the literature vary considerably and are probably method-dependent.

However, we are the first to report that RV pO_2_ is markedly higher than LV pO_2_ in control animals. It has long been known that the pO_2_ in the veins draining RV is higher than in those draining LV^23^ and that O_2_ extraction is higher in the LV than in the RV^23^. As RV oxygen consumption increases from rest to exercise, the initial effect is an increasing oxygen extraction (indicated by the falling venous oxygen tension). This increased oxygen extraction in the RV occurs with little change in flow when venous tension is above 10–12 mmHg. Conversely, in the LV increases in myocardial oxygen consumption result in negligible decreases in venous oxygen tension and large increases in coronary flow^23^. Our data nicely support these observations, indicating that indeed resting myocardial pO_2_ is much higher in RV than in LV. Higher extravascular compression in LV than in RV may be responsible for this phenomenon^24^.

Furthermore we showed here that RV pO_2_ was reduced by more than 40% in PH, while that in the LV was unchanged. This reduction correlated with both indices of reduced perfusion pressure (LVESP) as well as with indices of extravascular compression and oxygen utilization (RV wall stress). Thus it is difficult to determine, which of these factors was the main culprit here. Of note, no such correlation was found for LV pO2.

### Effects of ITPP on myocardial oxygen tension

Affinity of hemoglobin for molecular oxygen is regulated in human red blood cells by 2,3-diphospho-D-glycerate II (DPG), an allosteric effector of hemoglobin. ITPP binds to the same allosteric pocket, resulting in rightward shift of hemoglobin-O_2_ dissociation curve, especially under low O_2_ conditions^12^, which could explain its specific effects found in hypoxic tumor tissues^14^. Moreover, ITPP enters red blood cells through the Band3 transporter, which distribution is mainly restricted to erythrocytes, which is responsible for its red blood cell specific affinity^13,15^.

Here we showed that ITTP increased RV pO_2_ in the PH rats. What is interesting, RV pO_2_ in PH rats (18 mmHg) was still higher than LV pO_2_ both in control rats (15 mmHg) and PH rats (14 mmHg). However, acute administration of a single dose of ITPP improved only RV pO_2_ in PH rats (to 26 mmHg), without affecting LV pO_2_. This again stresses the fact that oxygen supply and utilization is differently regulated in RV and LV and what is acceptable for LV, is hypoxic for RV. This may be due to different density of mitochondria and capillaries between these two ventricles^10^, but factors responsible for these differences remain unknown.

### Limitations of the study

The study was performed solely in a model of MCT-induced PH, which is a limitation of the study. MCT is known to cause PH through injury of pulmonary arterial endothelium, but also causes local inflammation and fibrosis. Therefore our results require confirmation in other models of PH. We used only acute ITPP therapy, since the concept of chronic ITPP therapy was complicated by the fact that ITPP was also a potent stimulator of PTEN (phosphatase and tensin homolog deleted on chromosome ten), which could also contribute to its effects in the chronic setting^14^.

## Conclusions

We show that RV pO_2_ is markedly reduced in the rat model of MCT-induced PH and that a new agent, ITPP, that facilitates oxygen release from hemoglobin pecifically under hypoxic conditions, is able to restore it upon acute administration. This indicates that RV is indeed hypoxic in PH and oxygen supply and more broadly RV energetics can be an important target for PH therapy; ITPP may be one of such potential therapies. Future studies are needed to verify this hypothesis.

## Data Availability

All data are available at the Department of Clinical Physiology, Centre of Postgraduate Medical Education, Warsaw, Poland.
